# A New Cellular Architecture for Information Retrieval from Sensor Networks through Embedded Service and Security Protocols

**DOI:** 10.3390/s16060821

**Published:** 2016-06-14

**Authors:** Aamir Shahzad, René Landry, Malrey Lee, Naixue Xiong, Jongho Lee, Changhoon Lee

**Affiliations:** 1 École de Technologie Supérieure, 1100 Notre-Dame Street West, Montreal, QC H3C 1K3, Canada; mail2aamirshahzad@gmail.com (A.S.); renejr.landry@etsmtl.ca (R.J.L.); 2 Center for Advanced Image and Information Technology, School of Electronics & Information Engineering, Chonbuk National University, 664-14, 1Ga, Deokjin-Dong, Jeonju, Chonbuk 561-756, Korea; 3 Shanghai Key Lab of Modern Optical System, and Engineering Research Center of Optical Instrument and System, Ministry of Education, University of Shanghai for Science and Technology, No. 516 Jun Gong Road, Shanghai 200093, China; 4 Department of Business and Computer Science, Southwestern Oklahoma State University, Oklahoma, OK 73096, USA; 5 Department of Fire Service Administration, WonKwang University, Iksan 570-749, Korea; 6 Department of Computer Science and Engineering, Seoul National University of Science and Technology (SeoulTech), Seoul 01811, Korea; cryptography1@gmail.com

**Keywords:** cellular protocols and networks, intelligent sensor networks, supervisory control and data acquisition system, security issues, embedded protocol security, information analysis and visualization, Human Machine Interface, transmission flows

## Abstract

Substantial changes have occurred in the Information Technology (IT) sectors and with these changes, the demand for remote access to field sensor information has increased. This allows visualization, monitoring, and control through various electronic devices, such as laptops, tablets, i-Pads, PCs, and cellular phones. The smart phone is considered as a more reliable, faster and efficient device to access and monitor industrial systems and their corresponding information interfaces anywhere and anytime. This study describes the deployment of a protocol whereby industrial system information can be securely accessed by cellular phones via a Supervisory Control And Data Acquisition (SCADA) server. To achieve the study goals, proprietary protocol interconnectivity with non-proprietary protocols and the usage of interconnectivity services are considered in detail. They support the visualization of the SCADA system information, and the related operations through smart phones. The intelligent sensors are configured and designated to process real information via cellular phones by employing information exchange services between the proprietary protocol and non-proprietary protocols. SCADA cellular access raises the issue of security flaws. For these challenges, a cryptography-based security method is considered and deployed, and it could be considered as a part of a proprietary protocol. Subsequently, transmission flows from the smart phones through a cellular network.

## 1. Introduction

Supervisory Control And Data Acquisition (SCADA) systems are computer-based Industrial Control Systems (ICSs) employed to gather and analyze in real time for monitoring and control purposes critical information collected from diverse equipment. Due to the dramatic changes in Information Technology (IT), SCADA systems can be deployed, and their remote field devices controlled and monitored through wireless networks, which increases the potential to access, gather, and examine critical information for industrial automation [1,2,3,4]. With the evolution of wireless technology, the installation cost of wireless-based SCADA systems has been significantly reduced by up to 10% compared to wired network installations or wired alternatives and operates much faster than those alternatives. In addition, wireless SCADA systems can save the engineering costs often required for SCADA wired networks such as large-scale surveys, wire installation and maintenance. In particular, they provide increased transmission access to gather information from wirelessly connected field devices [5,6,7].

In SCADA-dependent sectors like the oil, gas and water industries, the data transmission occurs from remote devices that may be placed at a great distance which is why Ethernet-based wire line connections are not feasible. In a few other cases wired networks are not feasible due to the need to provide multiple access stations or are limited by the specific locations of sensors and equipment. In these cases, the wired access from the SCADA devices can be replaced and the sensor results carried via wireless networks which are often considered to be a cost-effective and time minimizing solution for SCADA systems [4,6,8,9]. In wireless SCADA systems, the information can be carried through private radio lines and satellite transmission, which have very different characteristics such as distance range, data rate and transmission time that are all distinguished by the required associated fees. For instance, in the case of private radio lines the built infrastructure and related costs are a one-time investment, while in case of satellite data transmission, payments accrue according to the service used (access) [6,10,11]. Another major difference between these two types of wireless transmission (*i.e.*, private radio lines and satellite) are their potential for coverage enlargement. Repeaters are commonly installed to extend the signal strength if the distance between the connected stations is excessive. Satellite transmission provides a large coverage range of about 22,300 miles (or more) over which signals can be transmitted, but there is no solution for users to extend the coverage area since only a limited number of service providers are authorized to do that. In SCADA satellite transmission, private radio lines are typically used to extend the coverage range for those remote sites which would not lay on a satellite’s planned coverage map to get data access to/from suitably equipped field devices. Traditionally, wireless SCADA systems are designed to follow a point-to multipoint network architecture in which the SCADA field devices, remote terminal units (RTUs) and PLCs are each programmed with unique system addresses. These unique addresses are configured and can manage the control site using a SCADA human machine interface (HMI). The master host at the central control polls the configured nodes via these unique system addresses and stores the response information in a history (or database), that can be installed and located on a separate computer system. The overall transmission is performed by employing industrial protocols such as DNP3, Modbus, and Fieldbus, and supported through the SCADA HMIs [10,11]. NetSCADA is a HMI and SCADA system developed by Bentek Systems [6], and designed to accommodate several industrial protocols with an embedded SQL database and access input/output tags and runtime packages, with minimal cost of ownership. The NetSCADA design is fully supportive of client/server applications over the internet and its object-oriented configuration allows users to manage multiple new remote sites and to replicate remote information in a minimal time (session duration) as required by a simple SCADA system HMI [6,10].

**Figure 1:**
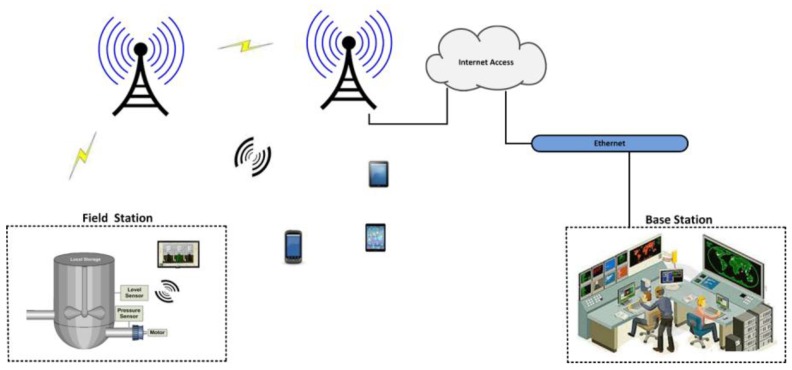
A cellular system.

With the substantial advances and adoption of wireless communication technologies in industrial automation, SCADA systems can also be accessed and monitored (or transmit) via cellular systems, which extends their productivity, transmission coverage area and transmission time [7,11,12]. For SCADA cellular transmission, cellular modems are used, which have several advantages compared to wireless local area network (WLAN) modems such as larger coverage mobility and more power to collect and transmit information without the restraints of wireless hotspots or Ethernet-based networks [11,12]. Cellular technologies like Code Division Multiple Access (CDMA) and Global System for Mobile Communications (GSM) are often used in the USA and other parts of the world and are deployed for SCADA systems or SCADA cellular systems. Cellular equipment like cellular modems is often used in cases that require coverage of larger geographical areas, with less consumption cost for satellite usage and other relevant technologies [12]. Figure 1 shows the general architecture of such a cellular system.

In a SCADA cellular architecture, the main controller or the host controller is at the top level and is designed to monitor and control remote equipment that could be sensors, actuators, and PLCs, which are locally designed, networked, and authorized to communicate through a cellular gateway, according to the main controller’s requests or commands. Typically, an application controlling server or cellular-based SCADA web server is installed and configured to control the whole remote networked site or overall SCADA cellular system grouped as “mobile originated and mobile terminated nodes” [12]. In the case of a mobile originated node, the connection is initiated from field devices that are connected remotely to the main host or main controller. In the case of a mobile terminated node, the connection is initiated and information is polled from the main controller (site), the networked field devices are intelligent for transmitting responses back to the main controller which usually involves equipment activity status, alarms and events. However, both the main controller and remote field devices are able to terminate the connection in the case of nodes (*i.e.*, mobile originated and mobile terminated nodes), commonly done at the web server [12,13,14].

To efficiently utilize cellular technologies such as CDMA and GSM, compatible cellular modems compatible with the service provider and its data plan are used. In-short, cellular modems are the main factors when deploying and using an efficient cellular system. Moreover, like other conventionally-based network modems, cellular modem technologies have also advantages such as compatibility with existing infrastructures that minimizes the cost, and extended cellular coverage, making them a significant alternative to wired networks, enlarging users’ mobility access, *etc.* Disadvantages include the need for control and support via service providers, limits, costly data plans and dependency on local or regional service providers in case of coverage increases, and transmission delays due to atmospheric effects [12,14,15,16]. Therefore, careful thought is required during the selection of any cellular modem technology and its required data plan, so that the chosen cellular modem has the necessary technical and update service support, and is convenient for the repeaters. Thus in important situations this technology has to extend the signal strength or coverage area, the service provider plans and their target sites coverage, and carrier selection are all important factors to be considered.

In recent years, industrial vendors and suppliers have become involved in accessing power systems and monitoring and controlling power distribution through cellular transmission. Based on their best practice outcomes and guidelines, SCADA industrial vendors and users have also examined the advantages that are to be taken into account to enable SCADA wireless (or cellular) systems [12,17]. For a robust and intelligent SCADA cellular system, the selection of the end-point devices is considered a big challenge, therefore the remote networked devices should be efficient and offer reliable wireless configuration, access, management and control, from sites located geographically anywhere, or from the designated control center(s). Moreover, the end-points or remote network should have robust persistence during a session and information delivery; reliable interoperation with industrial embedded protocols, like the Distributed Network Protocol (DNP3), Modbus protocol, Fieldbus, Transmission Control Protocol/Internet Protocol (TCP/IP), *etc.*; robust end-point built-in security and support for VPNs, IPSec, firewalls, *etc.*; reliable and solid cellular material for housing purposes; firmware with efficient wireless upgrading options, selection of wireless network equipment from a range of vendors with cellular expertise, and cellular connectivity via devices that use wireless rather than USB drives or other PC cards [5,10,17]. In [18], it was reported that in 2014, a total of 74% of identified users employed mobile technology and devices that provide fast and easier access to industrial or plant networks and thereby increase the productivity and profitability, and this percentage increased more in 2015. At the same time, security was identified as a potential and major concern, mentioned by over 50% of wireless technology users and more than 59% of mobile technology users [5,18,19].

As the cellular networks setups are configured and controlled using cellular modems, routers, access points, repeaters and gateways, the larger the network setup the more security issues will also be raised. Most cellular devices have built-in security solutions and security firewalls that are designed to protect the communication and wireless transmission over the internet. Most of the communications are dependent on wireless security protocols (*i.e.*, WPA, WPA2; and encryption and SSL). However, these security protocols have many security flaws that could easily weaken the security of networks and they depend on the cryptography protocols used [19,20,21]. To access the SCADA system information through the employed cellular system, the current study proposes a way to access industrial system (or SCADA system) information via cellular communication or cellular devices. The information is transmitted through secure channels established between the SCADA sever and the cellular device by a deployed cryptography (security) mechanism. To achieve the current study goals, the main objectives are as follows:

(1) This study models the SCADA DNP3 protocol which carries the information from field sensors to sub-controllers and from sub-controllers to the main controller (or SCADA server) through the encapsulation of DNP3 frames into the TCP/IP packets to transmit DNP3 payload over the internet.
(2) This study uses the DNP3 protocol payload design and its data link layer. The security is deployed before transmission of frames over the internet. For security, a cryptography-based mechanism, the AES algorithm, is deployed, which provides a secure way to transmit SCADA information over the internet access.
(3) This study uses DNP3 data link frame which is designed to carry and occupy 258 bytes of its single frame information and 34 bytes of cyclic redundancy code (CRC) information. These 34 bytes of CRC are not used in this study, since they are used for security design and implementation, and for keeping track of security information at both ends of the transmission.
(4) This study simulates a secure access mobile application that provides a direct way to access the real time SCADA information by connecting to the SCADA server. That is, the secure application is installed on registered and authorized cellular devices, and SCADA information will be accessed via this application login.

The rest of research paper is organized as follows: Section 2 describes the study of existing works in this area, and the existing development for mobile-based SCADA architectures. The DNP3 protocol and SCADA system are described in Section 3. In Section 4, a detailed system model is designed with formal proofs and security implementation is made that is considered as a protocol-embedded security. Performance results are determined, and relevant discussions are presented in Section 5. Section 6 provides the conclusions and future research directions.

## 2. Related Works

**Figure 2:**
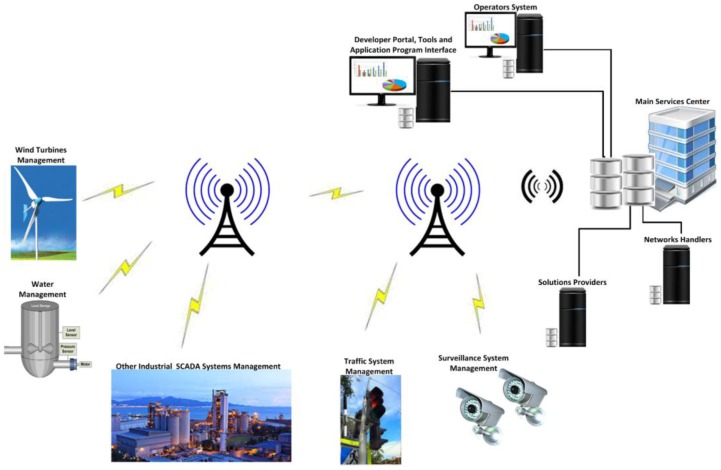
M2M cellular communications.

To reduce the network development and communication costs, SCADA uses cellular technology to control and monitor its industrial infrastructure and automation devices. Other software applications are mainly deployed to manage and operate the field-networked sensors, and other system components remotely, thought to be used for cellular communication in cost-effective ways [12,13]. To include further enhancements in SCADA automation and controls, a concept machine-to-machine (M2M) communication is used whereby facilities provide wireless access for the private- and public-based SCADA infrastructures to control and monitor the remotely located equipment in more efficient, reliable, and cost effective ways; moreover, SCADA architectures have been supported and designated for specific industries, but the M2M technology can also be deployed to manage them and is more advanced and efficient for systems, like industrial manufacture, IT and finance, public infrastructures, building monitoring and management, transportation systems, enterprise resource planning (ERP) and customer relationship management (CRM) systems, vineyards (fields) and farms, and others system management [22]. In conclusion, a variety of systems is supported by M2M technology, which could be networked, and located virtually at any geographical site. Like the diversity of applications supported by M2M technology, cellular communication is often used in SCADA industrial and manufacturing applications, public and private infrastructure monitoring and management, and agriculture and farm-based applications, and this opens new trends involving remote monitoring and controllers that have been linked with these organizations to provide more reliable, efficient, and optimal solutions that would enhance the organizational performance and profitability [12,22]. Figure 2 shows the M2M cellular communication for SCADA systems and for other systems.

The connectivity of SCADA cellular systems with remotely located devices provides several advantages such as easy installation and network setup, rigorous monitoring and control, cost effective operation, recognizable technology advances, and market competitive and manageable prices due to M2M technology [23,24]. Based on these advantages, in Stamford, the Stamford Water Pollution Control Authority (SWPCA) proposed and used a wireless SCADA system, in-which a “Smart Gateway” has been used to convert the transmitted packets into IEEE 802.3 wired Ethernet signals, and furthermore IEEE 802.1 is also employed as the wireless standard for communicating with dedicated industrial Access Points (APs) [25,26]. At the same time, SCADA wireless connectivity has some limitations, therefore, a few important factors should be considered before networking wireless systems or wireless field equipment, that are: remote power availability for wireless signals, and for sensors, power balancing corresponding to the desired information access, desired wireless converge ranges, and potential considerations against lost wireless links and connectivity. Thus, based upon these factors, wireless communication is suitable in the case of slow response sessions along with available power device or battery life that minimizes the cost of power wiring and other network setup and operational costs. Nowadays, the wireless network-based devices, such as Remote Terminal Units (RTUs), sensors, PLCs, power batteries, and other input/output terminals, are integrated as a unit that would be setup, uninstalled, and can be moved to new remote locations, according the requirements of industrial processes and their control and monitoring [23,24].

Kirubashankar *et al.* [11], proposed and employed a web-based automated control system for a water plant. The system design comprised a PLC and a SCADA control via computer system with the main objective of monitoring and checking the proper sequential flow of water during critical processes as water flew in and out from the plant, and to ensure the system security against web or internet attacks [27,28,29,30]. As the SCADA system information is monitored and controlled from an authorized client that could also add or modify the information, or send commands for processes in the plant, the overall system acts as a client/server platform [31]. Furthermore, the remote devices are connected via a network gateway, thus the plant information would be available over wireless channels; a day-to-day modern concept of M2M technology could also be applied that provided secure two-sided transmission, over wire/wireless networks, and GPRS and GSM cellular networks [31,32].

With the growing demand for technology in the area of SCADA systems and utilization of various advance hardware and software systems, SCADA/HMIs are required to manage the overall infrastructure(s) [33,34,35]. Typical SCADA system components (or devices) are designed to manipulate the SCADA communication on a lower bandwidth, which would restrict the availability of advanced multimedia information such as information in the form of audio/video and other high bandwidth information delivery. Due to the limitation of bandwidth, and also data delivery through data link layer, it is suggested to process the heavy multimedia audio/video information via a distinct channel, without interruption of the SCADA system and its employed normal delivery channel protocols [33]. Traditionally, SCADA communication was manipulated in a textual, or text based form, but with the enhancement and development of multimedia technology in various fields of IT, SCADA HMIs were also integrated with the advances in technologies for audio/video information, delivery and control [33,36,37]. SCADA systems mainly employ various proprietary protocols and vendors devices (or field devices) which softwares (HMIs) operate according to hardware specifications. Therefore, it is difficult for end-users to design and develop interfaces according to their demands; to design a user defined interface, a deep knowledge of the hardware is required [32,37,38]. As a consequence, while it might be difficult to design a SCADA/HMI without in-depth knowledge of the device information, is the end it is an absolute requirement from the users’ point of view [33,34,35]. Hence, the current research takes a step to employ a more convenient multimedia platform that visualizes the communication parts of the SCADA system.

A number of researches have been made to secure SCADA systems and their communications, and most of them are based on end-to-end security mechanisms, dependent on open security protocols such as, SSL/TLS, IPSec, and SSH, through the installation of security software such as firewalls, DMZs, intrusion detection and prevention mechanism and others [11,31,39,40,41,42,43,44,45,46,47,48,49]. A limited amount of literature has also described the trends in SCADA cellular communication security, but most of mentioned as proposed works, or developments in the initial stages [13,39,50].

The DNP3 user group has proposed a mechanism in conjunction with encryption, namely “secure authentication”, which provides security at the application layer, used at remote sites (or used by the sub-controllers) [51]. Through this mechanism, sub-controllers are able to verify where a transmission is coming from, and the operations are also secured against spoofing or replay attacks which are commonly seen to cause disruptions during sub-controller operations [11,50,51,52]. However, this mechanism is limited to provide security for the DNP3 protocol over the internet, and most of its security modules are still in the development phase [48,50,52].

**Figure 3:**
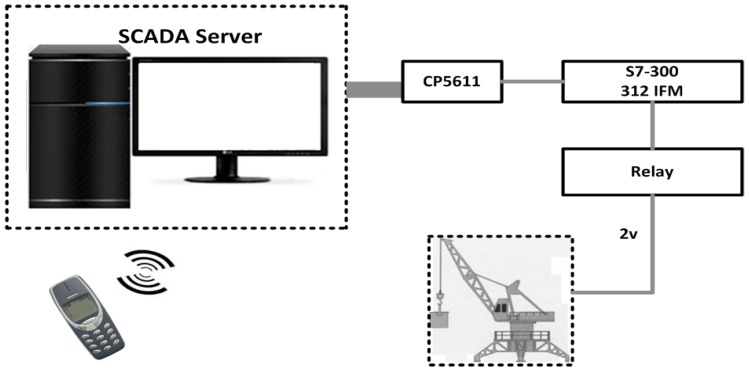
A Mobile-based SCADA application [53].

*Ozdemir’s Mobile-Based SCADA Architecture*: Ozdemir and Karacor [53], proposed a sample design to access real information from industrial automation systems via mobile phones. A sample application was designed as a cellular phone application, and a mobile phone was used as a host to monitor the designated parts of an “experimental prototype crane system.” To access and monitor the crane information using a mobile phone, general packet radio service (GPRS) or wireless application protocol (WAP) transmission were used, which offered significant advantages in increasing the performance without any effect on the SCADA system response-time. In the crane system model (in Figure 3), a PLC (S7 300-312 IFM), I/O card (SM334), a Siemens mobile phone (M50 with 228 Kb storage), and a computer system were employed. The sensors are used to move the crane in right-left direction, and for crane height measurement. Operation of the crane is controlled via two DC motors, and supervised by SCADA software. The communication between the server and test equipment is carried by the PLC which is also designated as a bridge for the purposes of data exchange. A MPI (CP5611) bus card is installed in the server which acts as a bus protocol to perceive the information that is exchanged between the server and PLC. A part of the system or the whole crane system is controlled by a graphical user interface (GUI) installed on a mobile phone with four main features as follows; (1) graphical animations, where the proposed prototype control values are received and converted into graphics; (2) the graphics animations that are displayed in the form of tables; (3) the alarm, that is available and repeated in an alarm panel in the prototype; and (4) remote control, where the system information is controlled remotely via separate pages defined in the GUI. For the mobile phone, a J2ME application program (JAP) is written in an integrated development environment (IDE), one studio 4, and tested in the Siemens M50 emulator. As a consequence, the information is retrieved by the user by means of a mobile phone and internet routing (techniques) through apparently automated on-line diagrams and through GSM technology. Basically mobile-users can control the operations of the crane system. In conclusion, this work provides little insight into the use of wireless cellular technology SCADA for industrial automation and to access field equipment readings (or information) via mobile devices and considerations of future prospects.

In SCADA-cellular based communication, the external information is transferred from a SCADA main center (or from a central controller) to a sub-controller (*i.e.*, a mobile device) via cellular services such as GPRS and WAP. At the same time, some internal communication and integration are required between the controllers that carry out the SCADA system information to make it possible to be monitored and controlled from the cellular phone [50,53]. Therefore, to view the desired operational information of the SCADA automation via a cellular phone, SCADA automation software is installed in a computer system or SCADA server. The software retrieves the real-time critical information from the field devices (or equipment) and employs control software or programs designed to register and control incoming information from the equipment to the server that will be stored in the history. However, the incoming information is totally dependent on the information required or requested from the server. Furthermore, the control program is also responsible for fetching the data from the history, and processing it towards a designated on-line web link. The sent information is stored on a web server and pushed toward the cellular phone through an active sever page (ASP) connection established between the SCADA system and the mobile phone. In the transmission, the cellular service GPRS or cellular standard WAP are used to pass the SCADA information to the ASP and further to the mobile device. In the mobile device, a user defined application such as a J2ME Application Program (JAP), is installed and designed to receive the information which is further stored, examined, and displayed, according to the user’s requirements [53].

Moreover, Short Message Service (SMS) services are provided by cellular systems and have been deployed in many sectors. Examples includes heart patients’ ECG readings, greenhouse monitoring and control, water level detection systems, crane systems, power systems, vehicle monitoring systems and temperature and humidity values that are monitored. The information is stored in a database and any corresponding alerts are transmitted to authorized users [12,48,54,55]. In another study, Raul *et al.* proposed a system in-which a TI MSP430F2274 microcontroller was used to sample sensors’ voltages and transmit the variations to mobile device, that could access, read, inspect and analyze the information through a web server that uses the Cinterion MC55iT GSM/GPRS terminal. However, SMS was restricted to cases of sudden changes occurring in normal data acquisition because the mobile phone was considered as a supervisory station with installation of LabVIEW software. This not only allowed users to view information, but also to monitor, analyze and control the entire system information [12].

## 3. The DNP3 Protocol and SCADA Systems

DNP3 is an open protocol originally designed and employed for electric industries, but which has gained popularity in other areas because of its efficiency and robustness, and has also been accepted and successfully deployed by water, oil, and gas industries, as part of SCADA industrial communication systems [27,54].

### 3.1. DNP3 Message Structure

DNP3 has four stack layers: application layer, additional pseudo-transport layer, data link layer and physical layer, and mainly uses TCP/IP and UDP that provide the communication facilities, or the way to communicate over the internet. In DNP3 message design, each layer performs distinct functions to ensure efficient and reliable communication over the transmission channels; the original size of messages built in the application layer is 2048 bytes that include a 2–4 bytes application layer header and 2046–2044 bytes for the user data or application service data unit (ASDU), in the cases of message requests and responses. Moreover, the pseudo-transport layer is able to carry 2048 bytes from the application layer, which will be further divided into eight parts of 249 bytes except the last divided part that will be of 247 bytes in length. The divided parts are also designated as user data blocks. The pseudo-transport layer adds one byte of header to each block (also called transport protocol data unit (APDU)) that will further use the data link layer [48,56]. More details of the DNP3 protocol layers are listed in Table 1.

The data link layer provides a reliable communication platform for the DNP3 messages while travelling over networks. CRC codes are also added to messages to provide an error detection mechanism. In DNP3, the data link layer takes transport protocol data units (TPDUs), and each TPDU is assembled as link service data units (LSDU). In the next stage, link protocol control information (LPCI) is added with LSDU bytes, also called link protocol data units (LPDUs) or link frames which are sized up to 292 bytes. The link header contains function codes that are used for initialization and testing operations of the logical link between the main controller and sub-controllers and vice versa, and has 10 bytes of header fields such as start, length, control, destination address, source address and CRC [56]. As described, the maximum size of each TPDU is up to 250 bytes with 1 byte of transport header, which can be easily fit within a link frame or LPDU. The link layer establishes the logical link to maintain a reliable communication between networked nodes over the physical channels. Moreover, transmission rules are defined that are applied to take actions for reliable communication and control bytes provide coordination, and also define the type of transmission between the participating nodes, as a master or a slave. The data link layer uses the FT3 frame format that defines overall frame structure, procedures or ways for communication and control byte information [48,56].

**Figure 4:**
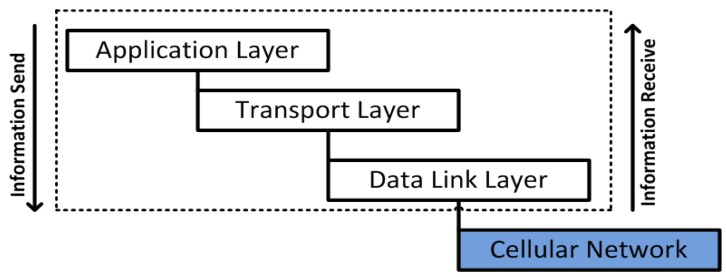
The Basic structure of the SCADA/DNP3 protocol.

The FT3 frame format was specified by IEC 870-5-1, and was the fourth format of IEC 870-5 among the other specified formats such as FT1.1, FT1.2 and FT2. The FT3 frame format, as applied in the data link layer, defines the overall link frame size. Its consists of a 10 bytes header, 32 bytes of CRC code , and optionally up to 16 bytes data blocks, while the last block or block 16 contains 10 bytes of data. The maximum size of LPDU is up to 292 bytes as specified by the FT3 frame format [56]. Figure 4 shows the basic structure of the SCADA/DNP3 protocol.

### 3.2. DNP3 for SCADA Systems

The DNP3 protocol has been considered an efficient protocol for SCADA industrial automation tasks and it is event-driven, and can be configured to exchange the information as input from a main controller and output (results) from networked field devices or sub-controllers [48,50]. The response would be a reply to a main controller poll, report, a current point’s values, alarms, and unsolicited responses from sub-controllers. According to the configuration of points and network setup, the main control frequently sends polls or integrity polls to sub-controller, which would also activate to respond with all the corresponding current points’ values in its DNP3 history. Furthermore, the DNP3 protocol design is able to provide communication over Ethernet and the internet, through encapsulation of the DNP3 frame into TCP or UDP packets, which make it possible to transmit over the internet. UDP has been considered as efficient due to its less packet overhead compared with TCP, therefore, it is of substantial value for SCADA cellular transmission. During communication, the DNP3 protocol has a built-in information logging facility, as the events generated, even if they may occur for only a few seconds, must be logged into the event queue (e.g., the MicroLogix 1400 PLC queue is designed to stored more than 6000 events) [50]. The data event would be generated continually, and logged into the queue until the established connection will not restore and applied in-case either the main controller does not get the response or a sub-controller does not get a reply to its unsolicited responses, in a session of several seconds. As a consequence, all information would have been observed and reported in the form of events, regardless of their frequency; all the DNP3 events that are generated from sub-controllers are time-stamped, and are also synchronized with the network time-stamp [27,50]. At the main controller site, the event data are received, which may have single or multiple changes, and the corresponding change information will be added to the history, with an original time-stamp in a millisecond format [50,56,57,58,59,60,61]. All this means the DNP3 protocol always provides a reliable and robust data transmission method for SCADA systems [48,50].

## 4. Proposed System Model and Design

Like traditional computer networks, there is also a requirement to access the remotely located industrial stations from a centralized station. Therefore, the best way is to employ a wireless technology such as a satellite system. Furthermore, the industrial processing system can access mobile phones via cellular networks. As a result, this study proposes a solution that could provide secure industrial access to mobile phones through cellular networks. In this section of our study, a proposed system model and its related definitions are presented. It is further used during protocol payload design, security implementation and payload transmission over a cellular network to mobile devices. For convenience, Table 2 summarizes the terminologies used in this study.

In a SCADA cellular system, a number of sub-controllers sb are employed and represented by a set $\text{SB}=\{sb_{i}|1\leq i\leq n\}$. Each sub-controller $sb_{i}$ is connected with the field devices or field sensors $fs_{i}\text{ FS}=\{fs_{i}|1\leq i\leq j\}$, where j is a limit that is designated for the field sensors $\;fs_{i}$ such as level sensor, pressure sensor, heater sensor, and cooling sensor; with specific k functional operation $\text{OP}=\{op_{i}|1\leq i\leq k\}$ that is connected with a SCADA system-compatible cellular gateway $cg_{i}$, and represented by a set $\text{CG}=\{cg_{i}|1\leq i\leq y\}$. It means that each sub-controller $sb_{i}$ uses a cellular gateway $cg_{i}$ that makes it possible for them to communicate over a cellular network. Therefore, $\text{CG}=\{cg_{i}|1\leq i\leq y\}\;\propto \text{ SB}=\{sb_{i}|1\leq i\leq n\}$. Each networked cellular gateway $cg_{i}$ transmits the information from a sub-controller to the main controller MC. In the overall system design, one SCADA cellular server or main controller MC is configured and networked over the Web (or internet) to control and monitor the remotely located sub-controllers $sb_{i}$. Moreover, the Cellular Devices (CDs) are authorized to access the SCADA system information through a main controller $sb_{i}\;$in a secure channel (SC). The information that is retrieved from MC to CD is denoted as $IMC_{id}$, where id is a unique identification that is allocated for the sub-controller $sb_{i}$. However, when information is delivered from MC to CD this id will be random, to avoid network attacks.

**Figure 5:**
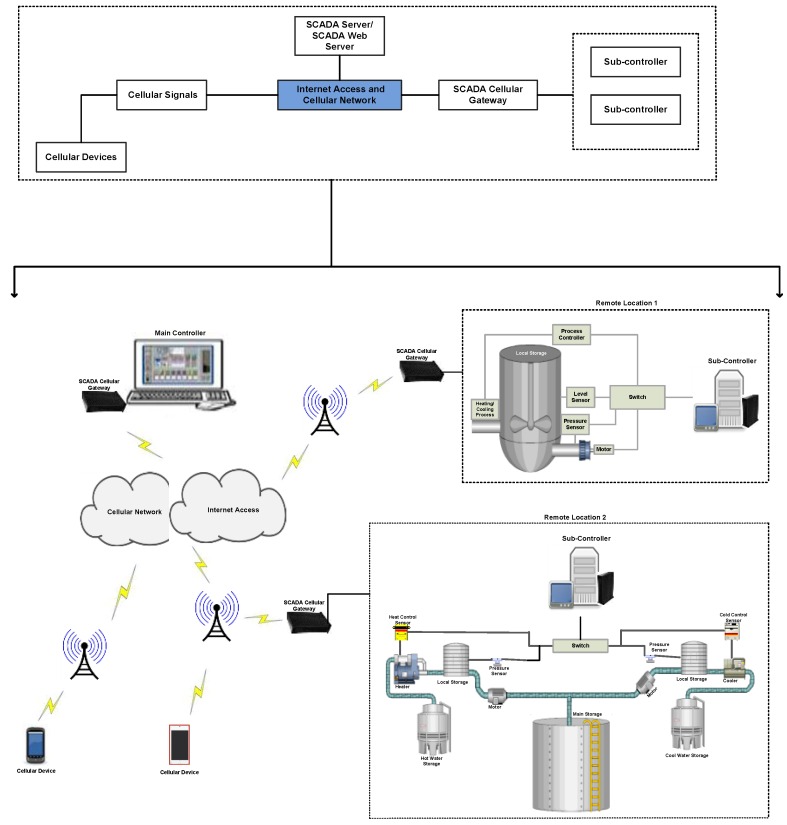
System design and setup.

The DNP3 is employed to configure the Main Controller (MC), Sub-Controller $sb_{i}$ and Field Devices$\;fs_{i}$. Each time when information, or message M is transmitted from $sb_{i}$ to MC, a secure channel (*i.e*., AES algorithm) is deployed and tested at the data link layer as a part of the DNP3 protocol. Meanwhile mobile users can access the system information by following these steps:

(1) First register as a user of the main server by filling out the registration form.

(2) After registration, an installer and AES security certificate is used to make a direct and secure connection with the SCADA server over internet access. Figure 5 shows the system components and their connectivity with each other in the form of a block diagram. For convenience the block diagram is further represented in a graphical form which is easier to understand.

### 4.1. Proposed System Definitions

Security is a big issue in industrial system (or SCADA system) data transmission. Therefore, to handle this issue, a development is proposed that is not considered as an external (or end-to-end) development but considered as a part of the protocol embedded security. For a secure SCADA cellular system, the DNP3 protocol is employed by employing its open library (and open source codes) without changing its internal stack and bytes flow:

#### 4.1.1. Definition 1 (System Controller Set)

A set of controllers $C_{s}$ in a system is denoted as $SC_{s}=\text{\{MC},\;(sb_{i}|1\leq i\leq n),\;{\left(CD\right)}_{i}\}\;$, where $sb_{i}$ is the number of sub-controllers and ${\left(CD\right)}_{i}\;$the number of mobile devices connected with a main controller. In the proposed SCADA cellular system, a set $\text{SB}=\{sb_{i}|1\leq i\leq n\}$ and ${\left(CD\right)}_{i}$ represents numbers of sub-controllers and mobile devices that are connected with a main controller, but for performance measurements we use only one sub-controller and one mobile device.

#### 4.1.2. Definition 2 (Protocol Assembled Bytes)

A set of protocol specified bytes J are received and assembled by employing the function $f(p_{\begin{array}{c} 1 \end{array}}$), and will be identical each time, such that :J≅Y. The data link layer uses transport protocol data unit (TPDU) bytes Y as user bytes. Bytes Y are assembled as a link service data unit (LSDU) $J_{d}$, or a user data d, as a part of DNP3 data link layer. The original size of each X is up to 250 bytes and the $J_{d}$ size is also identical.

#### 4.1.3. Definition 3 (Protocol Header Bytes)

A set of protocol header bytes $J_{h}$ are deployed by using function$\;f(p_{\begin{array}{c} 2 \end{array}}$), and added with protocol assembled bytes $J_{d}$, which formed the data link frame LF such that: $LF=f(p_{\begin{array}{c} 1 \end{array}},\;p_{\begin{array}{c} 2 \end{array}}$) =$\;(J_{h}+J_{d})=\;J_{\left(h,d\right)}.$

Here, the protocol header bytes $J_{h}$= [$J_{h\left(0\right)};J_{h\left(1\right)};J_{h\left(2\right)};J_{h\left(3\right)}]$ = [Start; Length; Control; Destination address; Source address] are deployed and added with protocol assembled bytes $J_{d}$, and a data link frame LF is formed. Every time, the maximum size of LF will be identical as 258 bytes, counting the CRC bytes.

#### 4.1.4. Definition 4 (Security Bytes)

Given and computed LF, LF=$J_{\left(h,d\right)}$, the security functions: f(Ey)and f(Dy) are deployed for security development. At the data link layer, the encryption function f(Ey) is deployed on computed bytes $\;J_{\left(h,d\right)}$, and the decryption function f(Dy) will be deployed at the target side. In case of mobile access, the security is installed, checked and tested via an AES-based security certificate. However, this development is open to deploying and testing other security algorithms from cryptography.

#### 4.1.5. Definition 5 (Security Development Controller)

For $\;J_{\left(h,d\right)}$ bytes and security development, additional functional bytes FB are added which help control and manage the security development. In security design and development, functional bytes FB are required that are employed to control and manage the overall manipulation of security development. We designed these functional bytes as Security Development Controller (SDC) bytes that define a distinctly significant meaning for the security development. The SDC defines and contains a total of 34 bytes of Cyclic Redundancy Code (CRC) from the data link layer. We have not used the CRC technique, but we have utilized these bytes (or 34 bytes), in our proposed work for security development. In SDC, each contained byte defines a significant meaning and was used during the whole security development process. The functional details of SDC bytes are given in ascending order in Table 3.

#### 4.1.6. Definition 6 (Device Registration, Authentication and Authorization)

The CDs are required to register an authorized SCADA cellular system user. For this, CD information is registered at the main controller MC and a device installer DI and a secure certificate SCT are used to access the information of the SCADA cellular system.

In the section below, formal proofs (*i.e.*, Postulate 1 and Postulate 2) are employed that validate the proposed system design and its deployment. Moreover, Postulate 1 is employed to validate the proposed system design and its deployment when communication occurs between a sub-controller and main controller and *vice versa*. On the other hand, Postulate 2 is employed to validate the proposed system design and its deployment when communication occurs between a main controller and a mobile device and *vice versa*.

### 4.2. Security Implementation and Byte Flow

In DNP3, the data link layer assists either connection oriented or connectionless transmission and provides a consistent link between sender and receiver over a physical channel [56]. Due to the specifications and services provided at the data link layer (during establishment of connection and address assignment), the percentage of attack detection (or abnormalities) is high and more harmful, compared to other DNP3 protocol stacks (layers) [10]. The data link layer also employs the CRC technique to detect the errors during transmission of bytes over the physical links which are established between SCADA nodes. However, this technique has several limitations and does not provides security against attacks [10,31].

#### 4.2.1. Postulate 1

A data link layer frame LF is constructed, security is implemented, and transmitted and received over the SCADA cellular system (Link Frame LF, numBytes nB, security DevelopmentController SDC, stackFlow SF, Encryption Ey, Decryption Dy)

The bytes X are received from the upper layer and are assembled as link service data units (LSDUs) $\;J_{d}$. The J is limited (Iim) and is similar to X (size). The assembled $J_{d}$ bytes are computed without CRC bytes. Such that:

(1) $$\forall X≅\forall J\Rightarrow J_{d}$$

For a link frame, the fields are: start (2 bytes), length (1 byte), control (1 byte), source and destination addresses (4 bytes) are computed, as part of the link header, and designated as $J_{h}$.

(2) $$J_{h}\Rightarrow J_{h}.Comp\left(J_{h\left(0\right)},J_{h\left(1\right)},J_{h\left(2\right)},J_{h\left(3\right)}\right)$$

By adding the link header $J_{h}$ with $\;J_{d}$ the link frame LF is formed. Each LF size is limited to 258 bytes (without CRC bytes), so in a case where more bytes are required then multiple LFs will be constructed:

(3) $$\Rightarrow {\left(J_{h}+J_{d}\right)}^{n}=\overset{n=limit}{\underset{k=0}{\sum }}\left(\begin{array}{c} n\\ k \end{array}\right)J_{d}{}^{k}J_{h}{}^{n-k}$$

Here, the limit shows the maximum size of each link frame LF. As a consequence, if $Comp\left(J_{h}+J_{d}\right)\neq 0$ then there is single LF; if $Comp{\left(J_{h}+J_{d}\right)}^{n}$ then there are multiple LFs; and if $Comp\left(J_{h}+J_{d}\right)=0$ then a link header is transmitted. Furthermore, the cryptographic AES algorithm is deployed on a computed frame LF before transmitting it to the cellular network:

(4) $$f\left(Ey\right)\;=\;M=Ey\left\{Comp\left(\;J_{\left(h,d\right)}\right)\right\}\;||\;SDC.\;update\left(bytes\right)$$

Message M is deployed by the computer with the AES encryption function f(Ey) and the corresponding information is updated within the security development controller (SDC). The AES algorithm uses the same key to perform encryption and decryption functions. In the proposed security development, the AES key or secret key is shared securely between the main controller (MC) and sub-controller (sb) before performing any encryption/decryption functions. Upon receiving the message M at the main controller side, the deception is performed by employing the function f(Dy). The authentication and confidentiality of message M will be evaluated to understand whether the shared secret was deployed successfully, such that:

(5) $$f\left(Dy\right)\left[f\left(y\right)\right]=f\left(Dy\right)\left[M\right]=f\left(Dy\right)[\;Ey\left\{Comp\left(\;J_{\left(h,d\right)}\right)\right\}]\;||\;SDC.\;update\left(bytes\right)$$

After the decryption process, the $J_{\left(h,d\right)}$ bytes are reformed. Furthermore, the $J_{h}$ bytes are separated from the link frame LF and $J_{d}$ are reformed and assembled into upper layer bytes (transport layer bytes X and application layer bytes Y), as a part of the DNP3 stack flow SF and are viewed by the SCADA system interface.

#### 4.2.2. Postulate 2

The information that was received from the sub-controller sb, is further manipulated by the main controller MC, and will be accessed by the cellular device CD (Request R, Connection C, deviceLogon DL, mainControllerAuthentication MCA, secureSession SS, refreshSecureSession RSS).

The mapping function is:

(6) $$f_{MP}:{\text{CD}}_{\left(R,C,DL\right)}\to {\text{MA}}_{MCA}\left({\text{CD}}_{\left(SS,RSS\right)}\right)\Rightarrow {\text{CD}}_{\left(R,C,DL\right)}\to {\text{MA}}_{MCA}\to \text{SS}\to \text{RSS}$$

⇒CD represents the mobile device that is being requested R for a connection C from the main controller MC.

⇒DL: a registered cellular device RCD is logged on via a Device Installer DI and passes the Secure Certificate SCT, to provide direct access to the main controller MC.

⇒MCA( ): upon a cellular device CD request R, the main controller MC will make the device authentication DA and provides the device authorization DAU to the cellular device CD.

⇒SS( ): after authorization, a Secure Channel Session SCS is created by employing the AES algorithm, in which the cellular device CD is authorized and can view the information as required. In SCS, the number of bytes NB flows continuous ly in sequential fashion and the cellular device CD is allowed to view this sequential Byte Flow BF.

⇒RSS ( ): if required to refresh the session or in case the existing AES session key has expired, a new session will be created based on mutual agreement (*i.e.*, MA and CD) and by encrypting the existing session key with new session key. Each time a session is refreshed, a similar encryption process will be deployed to avoid any unknown entities.

Each time a mobile device is logged on DL by the SCADA cellular installer and is passed the AES secure certificate SCT, and authenticated as an authorized and registered device of the SCADA cellular system, a connection called SS is established between the cellular device CD and main controller MC. The connection that is established through SCT is limited for a specific session and will be refreshed in accordance to the cellular device’s  CD request R and the mutual agreement MA.

## 5. Performance Results and Discussion

Nowadays, significant enchantments have been implemented in terms of communication that tend to facilitate easy access for users, like enhancements in computer networks. SCADA industrial automation can also be accessed and controlled by the use of various wireless-based electronic devices, such as laptops, tablets, and mobile phones. Tremendous enhancements have been made in cellular mobile technology, including cellular phones designs, in terms of their hardware and software design, to access and communicate over the internet via wired/wireless LANs/WANs [13]. Through these enchantments and effectiveness, cellular phones are also able to access, monitor and control almost all automation features of SCADA systems, through cellular networks, such as GPRS, GSM 2G and GSM 3G. In a SCADA cellular system, the remote networked sensor devices are configured to transmit information or sample data to authorized devices, at specified regular intervals, to the cellular gateway that is designated and coupled to the cellular system through the internet access [1,2,3,4,13,14].

The proposed work uses the SCADA cellular platform, in which cellular GSM-based communication is used to access and monitor the SCADA real time remote automation and processing via cellular phones. To achieve the desired goals of the propose work, a simulation-based SCADA water-pumping system networked with sensors and other field equipment [31], has been employed, where the information is acquired, points are sampled and transmitted to a cellular network that is a pathway by which information will be accessed by cellular devices (*i.e.*, Android phones and I-Phones).

In this study, the SCADA/DNP3 protocol as most prominent SCADA system protocol due to its worldwide use, despite its security shortcomings. The DNP3 designed protocols lack security features and with the used of modern information technology, they have has been connected with the internet via proprietary protocols such as TCP/IP, UDP and others [10,61]. As a consequence, a new secure development protocol is proposed, which is not considered as an end-to-end development, but rather considered as a part of the SCADA/DNP3 protocol. As explained, security is developed in the framework of the DNP3 data link layer, where 34 CRC designated and occupied bytes were used which finalizes the proposed security development.

### 5.1. Employed Experimental Setup and Configuration

In the overall SCADA cellular system, the SCADA server or main controller is superior and controls the system. The remote stations are networked to perform the operations of field devices (or sensors) and are also configured to send continuously the measured points according to the main controller’s commands. In this study, the remote users such as the cellular devices are allowed to access the SCADA system information via the SCADA main controller over internet access, but are limited to accessing the information directly from sub-controllers, to avoid security issues. To access the SCADA information, the users or devices have to be registered or will have to be registered with the SCADA main controller, and the user registration process is accomplished in a secure and usual manner which includes the details of the user’s full name, identification number, cellular device brand, type, company name, SIM card registration number, and other information. After registration, an installer is provided, and configured inside the mobile device and a secure cryptography certificate was also installed, which binds the user registered details with the SCADA server, or/and *vice versa*. When the user (or mobile user) is logged on, a direct connection will be established which provides a secure channel in which the SCADA system data will be continuously delivered to the mobile device(s). However, the information access depends upon the restriction policies and limitations according to the user registration agreement.

### 5.2. Experimental Design and Information Access

**Figure 6:**
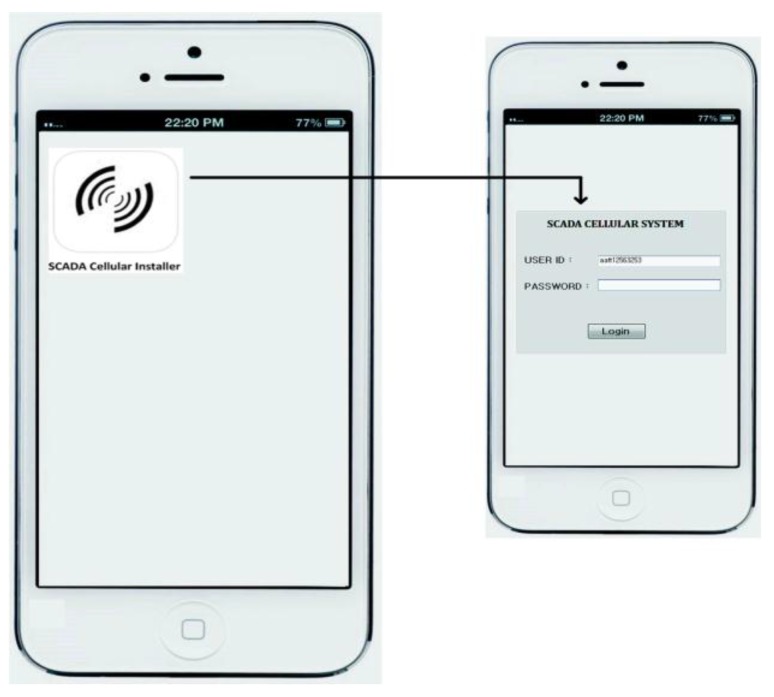
The Cellular device secure login.

**Figure 7:**
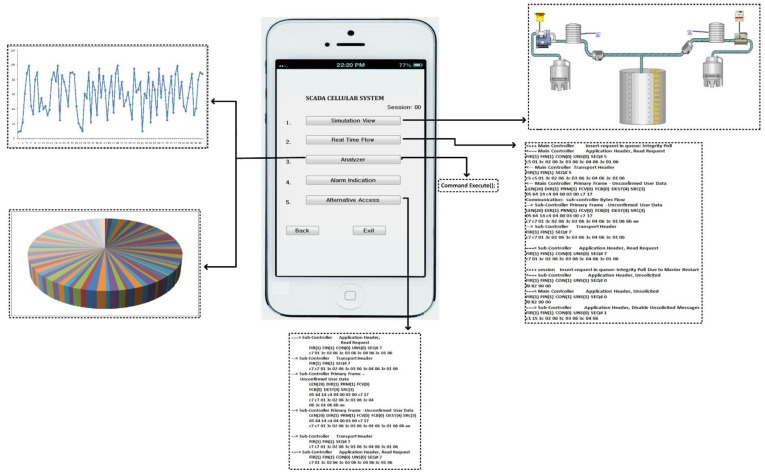
The SCADA cellular interface.

In Figure 6, whenever the user logs in via his/her mobile device (*i.e.*, Android or I-Phone), the connection will be directly made to the HTTP server toward the SCADA Web server in the presence of internet access. After the verification process by the main controller, a secured channel access is established and permission is granted to the mobile user, and as a result the mobile user will be able to access the SCADA information, while accessing the interface that has options to access the desired information of the SCADA system. In Figure 7, a user authorized interface is shown in a mobile device, which shows the user enabled options (buttons) to access the desired information of the SCADA system.

For SCADA information access, the given interface provides options (or buttons) such as simulation view, information flow, analyzer, alarm indication, and alternative access, for a mobile user to access and get the real time information of the SCADA system. In Figure 7, each mentioned option in interface provides a specified functional access for the mobile user:

(1) *Simulation view*: by enabling this option a mobile user would view the water pumping station in a graphical presentation which shows the overall system operation with its components such as water storage tanks, employed sensors, and other features.

(2) *Real time flow*: by enabling this option the transmission flows between the sub-controller and main controller which would be seen by the mobile user such as the main controller request commands and sub-controller responses.

(3) *Analyzer*: the mobile user is also authorized to view the overall SCADA system performance (past and current) in the form of graphics such as line graphs, bar graphs, column graphs, *etc.* Mobile users simply select the option, date, time and available graph options, as part of the analyzer option, and can view the SCADA system performance.

(4) *Alarm Indication*: this is a special function used by the mobile user. In the SCADA cellular system, transmission flows could be in accordance to the normal point settings, so in case an abnormal transmission flow (*i.e.*, abnormal points or/and network attack cases), the mobile user will alter the SCADA server via an alarm indication. However, the SCADA cellular system setup is intelligent enough to detect the abnormal transmission flows or measurements that may occur in the overall system, such as in server to sub-controllers pathway and *vice versa*.

(5) *Alternative Access*: this option will only be enabled in case the main server is functionally disabled, *i.e.*, during the time of system update, a alterative access will authorize mobile users to access the SCADA system information directly from the remote site or from a sub-controller. However, this will be a case that should occur very rarely.

### 5.3. Results and Analysis

**Figure 8:**
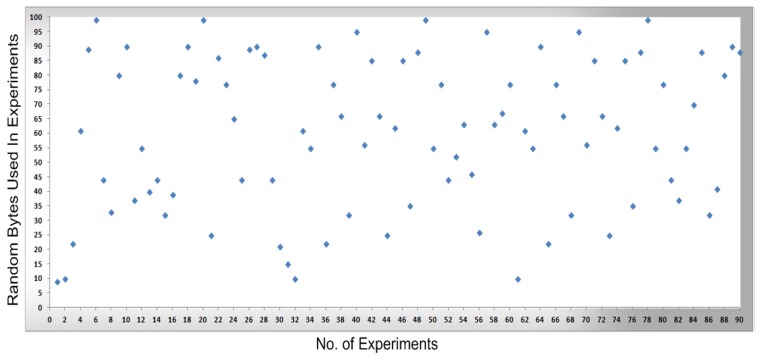
The transmission of bytes from a sub-controller to a main controller.

**Figure 9:**
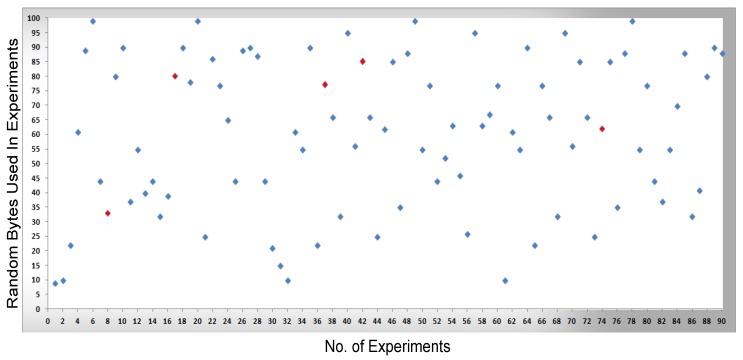
The transmission of bytes received at the main controller and transmission errors

**Figure 10:**
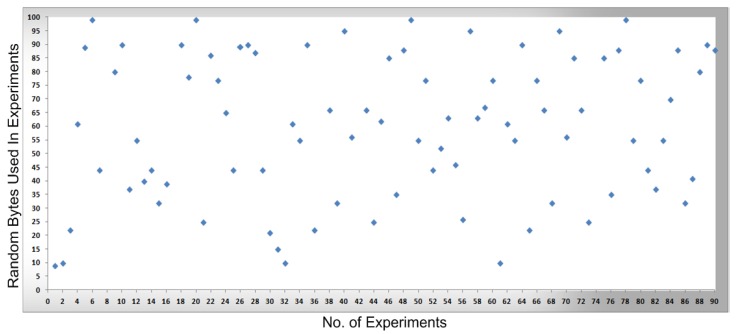
The transmission of bytes sent from the main controller to a cellular device.

**Figure 11:**
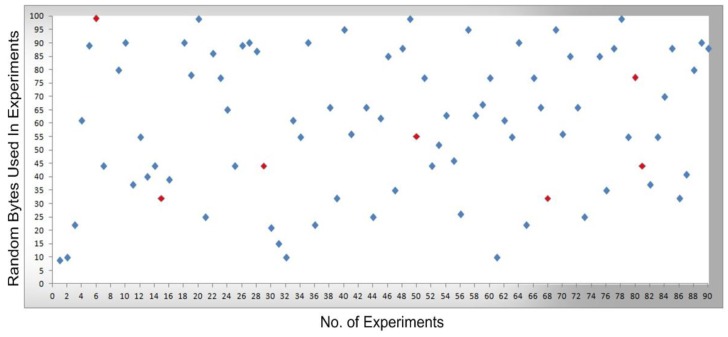
Bytes received at the cellular device and transmission errors.

In Figure 8, Figure 9, Figure 10 and Figure 11, measurements were conducted based on the system design and setup and the axes. The x-axis shows the total number of experiments performed to conduct the measurements and the y-axis shows the random sized bytes which are transmitted in each number of experiments. Furthermore, Figure 8 shows the successful SCADA/DNP3 random sized packets transmission flows (e.g., without any transmission errors or packets loss) which are transmitted from the sub-controller to the main controller and labeled with blue markers, while Figure 9 shows the sub-controller transmitted packets received at the main controller. In Figure 9, a few markers were labeled in red color which shows the packets that were lost or not received at the main controller side, so in Figure 8 and Figure 9 the blue markers designate successful transmissions and the red colored markers designate packets lost in transmission from the sub-controller to the main controller.

In Figure 10, the packets are transmitted (e.g., without any transmission errors or lost packets) from the main controller to the cellular device, while Figure 11 shows the result of packets received at the cellular device, where a few received packets are labeled in red color which shows the packets that lost or not received by the cellular device. In short, in Figure 10 and Figure 11 the blue markers designate successful transmissions and the red colored markers designate for those packets that were lost in transmission from the main controller to the cellular device.

By comparing the   performance measurements in the successful transmission flow case (without lost packets) and unsuccessful transmission flow (with lost packets), the overall system performance is efficient. In the performance Figure 8, Figure 9, Figure 10 and Figure 11, the number of performed experiments are labeled on the x-axis and the transmitted random size packets are labeled on the y-axis; in transmission between the sub-controller, main controller and cellular device, originally the packets were variable in sized but to compute the sequential transmission flows, the transmitted packets between the controllers, are shown in Figure 8, Figure 9, Figure 10 and Figure 11 were of the same size.

### 5.4. Significance and Limitation of Study and Future Recommendation

The current development is new in terms of providing mobile users with secure access to SCADA systems, where the security development is not be considered as an external development, but rather implemented as an part of the SCADA/DNP3 protocol security. Therefore, the transmitted packets (or payloads) will be secured while encapsulated in other open protocols (such as TCP, UDP, GSM, GPRS, WAP and others) and travelling over unsecure media, such as the internet and cellular networks. This overall study and its development is based on simulation works on a water pumping system, SCADA/DNP3 protocol design and security development at the data link layer, SCADA cellular system and its access to mobile users, and others. In the near future, this simulation work will be implemented in a real environment.

## 6. Conclusions

With the technology progress in cellular systems, there are also demands to monitor and to control in real time industrial systems (or SCADA systems) using personal mobile phone(s). Typically, SCADA systems have employed proprietary protocols to carry out their communication between the sensors/devices that are configured and networked as process automation systems. The situations where several sensors that are located at many places must be interconnected to a main controller, are resolved by means of non-proprietary protocols. Thus, complicated configurations and connectivity services are required to process the proprietary protocols that generate information to and from non-proprietary protocols. As a consequence, in the proposed study, the bytes (or information) of a SCADA system is generated by the employed DNP3 protocol, and the proposed security solution is deployed at the data link layer before transmitting to open networks and/or cellular networks. A high percentage of security flaws usually occur in the data link layer of the DNP3 protocol due to its operations and connection orientation over physical channels. After passing the frames to a cellular network, these frames are further accessed by cellular devices with monitoring and control facilities that could act as an authorized user of the SCADA cellular system. The authorized cellular device could then view, monitor and control (*i.e.*, provide alarm indications), the SCADA system information according to the premises. To manage and provide a secure pathway in-between the controllers, a cryptography mechanism was deployed that should provide significant security strength for SCADA cellular system. This overall study is based on simulation works, and is remarkable in terms of SCADA cellular access and its corresponding security enhancements. In the near future, this proposed work (or appropriate simulation work) will be designed for implementation in a real environment.

## Figures and Tables

**Table 1:** The Description of DNP3 Layers.

DNP3 Layers	Header Length	User Data Length	Description
Application layer	2–4 bytes	2044–2046 bytes	2 bytes of header and 2046 ASDU bytes in the case of message request. 4 bytes of header and 2044 ASDU bytes in the case of message responses.
Pseudo-transport layer	1 byte	249 bytes	1 byte of header is added with each data blocks in cases, message requests or message responses.
Data link layer	10 bytes	250 bytes	10 bytes of link header is added with each upcoming TPDUs, in-cases of message requests or message responses. Moreover, 32 bytes cyclic redundancy checker (CRC) code is used for error detection.

**Table 2:** Terminologies for System Model and Design.

Notations	Descriptions
$sb_{i}$	Number of Sub-controllers.
$\;fs_{i}$	Number of Field Sensors.
$cg_{i}$	Number of cellular gateway
$IMC_{id}$	Information that retrieved from Main Controller MC to Cellular CD, with unique identificationid.
$op_{i}$	Number of k functional operation.
$SC_{s}$	Ccontrollers $C_{s}\;$ in a system S.
J≅Y	Uppen Layer Bytes.
$FL=J_{\left(h,d\right)}$	Link Frame LF is fromed by adding of assembled bytes $J_{d}$ and header bytes $\;J_{h}$.
[$J_{h\left(0\right)};J_{h\left(1\right)};J_{h\left(2\right)};J_{h\left(3\right)}]$	Protocol header bytes, such that: $J_{h}$ = [$J_{h\left(0\right)};J_{h\left(1\right)};J_{h\left(2\right)};J_{h\left(3\right)}]$ = [Start; Length; Control; Destination address; Source address]
$f(p_{\begin{array}{c} 1 \end{array}}$)	Bytes Assembling function.
f(Ey)and f(Dy)	Cryptography functions: Encryption (Ey) function f(Ey) and decryption (Dy) function f(Dy)
DI	Device installer DI
SCT	Secure certificate
SDC. update( )	Bytes Updated inside security development controller (SDC).
MCA	Main Controller Authentication
RSS	Refresh Secure Session RSS
SS	Secure Session
DL	Device Logon,

**Table 3:** Security Development Controller.

Security Development Controller		
Field’s Name	Length	Description
External addresses	4 bytes	Four bytes are defined for external source and destination addresses. The data link layer provides and maintains a reliable logical connection between the SCADA/DNP3 master and slaves (or between the master unit and sub-controllers), and addresses are also specified by this layer. In a few cases, encrypted data link layer frames (or LPSUs) might not verify at the receiver side, this is because the data link layer header (or LPCI) is also transmitted as hidden bytes. Therefore, external addresses are defined and transmitted with encrypted information. The sub-controller also verifies the message contents (or encrypted header information) corresponding to external address information [14,18,48].
Security checker	1 byte	Security checker function is defined that ensures the security development via cryptography, and also generates an exception in case of security failure (or unsuccessful deployment).
Acknowledgment	1 byte	One external byte is employed for acknowledgment purposes.
Critical/non-critical	2 bytes	Two bytes are defined as critical or non-critical bytes which check the normal and abnormal flow of traffic.
Selected Method	1 byte	The selected method (or changed method) function is employed which will dynamically change the security method or security algorithm. For example, in this research three algorithms (AES, RSA and SHA-2) are employed to enhance the security of the SCADA/DNP3 system. The RSA algorithm is not appropriate for SCADA/DNP3 broadcasting due to number of keys required in transmission, but it is appropriate for unicasing [14,15,16,17,18], therefore, the algorithm selection is made based on communication requirements or/and requirements of algorithms from the arena of cryptography.
Key sequence	1 byte	Keeps the information of cryptography keys in sequential order during generation and distribution.
Optional	1 byte	An optional function is deployed that verifies the contents of messages before transmitting them to an open network,
User bytes controller	4 bytes	Four bytes are deployed that keep track of the data link layer byte information such as the number of link service data unit (LSDU) bytes from the upper layer, LPDU bytes and security computation bytes.
Dynamic storage, padding and future use	16–34 bytes	These bytes are occupied by dynamic fields designated as dynamic storage and dynamic padding. Dynamic storage allocated the bytes to the existing fields, if they are required and in-case a new function will be added. The security development has been made and remaining bytes are padded with zeros.
